# Analysis on the trend of AIDS incidence in Zhejiang, China based on the age-period-cohort model (2004–2018)

**DOI:** 10.1186/s12889-021-11050-x

**Published:** 2021-06-05

**Authors:** Zhenzhen Lu, Weidong Ji, Yi Yin, Xinye Jin, Lu Wang, Zhongjie Li, Ning Wang, Kai Wang, Zhihang Peng

**Affiliations:** 1 Department of Epidemiology and Biostatistics, School of Public Health, Nanjing Medical University, Nanjing, 211166 Jiangsu China; 2 College of Public Health, Xinjiang Medical University, Urumqi, 830011 China; 3 National Center for AIDS/STD Control and Prevention, Chinese Center for Disease Control and Prevention, Beijing, China; 4 Department of Medical Engineering and Technology, Xinjiang Medical University, Urumqi, 830011 China

**Keywords:** AIDS, Age effect, Period effect, Cohort effect, Prediction

## Abstract

**Objective:**

To predict the trend of AIDS in specific age groups and to determine the objective population for AIDS screening, this study explored the three transmission routes and characterized each patient group using the APC model based on the whole, local, and immigrant populations in Zhejiang, China.

**Methods:**

The data recruited in this paper was obtained from the national Comprehensive AIDS Prevention and Control Information System - Antiviral Therapy Management database and the Chinese Disease Prevention and Control Information System and the Statistical Yearbook of Zhejiang, China. An APC model was used to estimate the impact of age, period, and cohort on the incidence of AIDS, as well as to predict the AIDS incidence in specific age groups based on different sexes with different transmission routes.

**Results:**

The AIDS incidence peaked in males aged 20–35 years; the incidence of males was higher than that of females due to the impact of period; obvious cohort effect was observed among the immigrants. In the whole and local populations, the incidences of males in all age groups and females in both the 35-year-old group and the whole age group were predicted to increase sharply in 5 years. In the immigrant population, the AIDS incidences in both sexes in all age groups were expected to increase significantly in 5 years. Under the influence of period, the incidence of AIDS via homosexual transmission in the whole population and the local population increased and remained stable after 2015. At the same time, the incidence of AIDS transmitted by homosexual and heterosexual routes in the immigrants also showed an increasing trend.

**Conclusions:**

The results elucidate that there are sex differences in AIDS incidence, and the incidence of AIDS through various transmission routes in all groups is predicted to exhibit an upward trend in the 5 years to come. Effective intervention strategies should be developed and implemented by the public health departments in Zhejiang to control the epidemic of AIDS.

**Supplementary Information:**

The online version contains supplementary material available at 10.1186/s12889-021-11050-x.

## Highlights

1. An APC model was applied to different populations in Zhejiang Province of China to analyze the incidence trend of AIDS via three transmission routes in each group.
2. The incidence of AIDS among 20–35 years old men and women in each group was significantly affected by age effect.
3. The incidence of AIDS and the increase rate in men were both higher than that in women.
4. The AIDS incidence in the immigrant population was expected to increase significantly in the next 5 years, which differs from the other populations.
5. The incidence of AIDS was predicted to increase in the next 5 years.

## Background

Acquired immunodeficiency syndrome (AIDS), a global health crisis caused by human immunodeficiency virus (HIV) [1], can affect and then destroy T lymphocytes of the human immune system, making the body unable to resist infections and diseases and even leading to opportunistic infections and deaths [2]. The main routes of AIDS transmission are needle sharing among drug users, commercial sex between sex workers and clients, unsafe blood donation and vertical transmission from mother to child. By 2018, 77.3 million people had been infected with HIV and 35.4 million had died of AIDS-related diseases worldwide. Although new HIV infection cases fell by 36% between 2000 and 2017 and the number of HIV-related deaths by 38%, the number of people living with HIV increased by 14% between 2010 and 2017, indicating that the HIV/AIDS epidemic is still on the rise [3].

In China, the first case of AIDS was an Argentinean man travelling to China in 1985. The first local case of HIV infection, found in the same year, was a hemophiliac in Zhejiang Province in eastern China [4]. Since then, HIV/AIDS has spread throughout the country [5]. By July 2017, about 728,270 people in China had been infected with HIV/AIDS and 223,798 had died of AIDS-related diseases [6]. Currently, 780,000 people are estimated to be living with HIV/AIDS, and 21.0% of them are migrant population [7]. The high risk of HIV infection among migrants can be attributed to the following social, economic, and political factors: unavailability of permanent household registration (hukou), marginalization, and insufficient health education, public health services and health insurance [8]. Although studies have shown that males have a higher incidence of AIDS than females in many countries [9, 10], the sex difference is not significant in the HIV/AIDS population in China. Currently, few studies have focused on HIV/AIDS prevalence and heterogeneity among the local population and migrant population in China. Therefore, a better understanding of the sex differences in HIV/AIDS among the local and immigrant populations and the differences in the HIV/AIDS incidence through different routes of transmission is crucial for AIDS prevention and control in China.

APC model, a statistical method based on Poisson distribution, can analyze the birth cohort factor and period factor at the same time, while the traditional modeling technology can only focus on one of the factors. APC model is widely used in research about incidence and mortality of cancer [11–13], but it is rarely applied in studies on chronic infectious diseases such as hepatitis B, tuberculosis and AIDS. The application of APC model in the study of chronic infectious diseases in China will be of great significance for disease prevention and monitoring. Therefore, for the study of AIDS incidence, the APC model can better simulate the patterns and relationships, and explore the impact of age effect, period effect and birth cohort effect. As far as we know, we are the first to apply the APC model to the study of AIDS incidence.

Zhejiang is an economically developed province along the southeast coast of China. By the end of October 2018, there had been 26,575 HIV/AIDS patients living in Zhejiang province. Sexual contact is the main route of HIV infection for these people, among whom 38.4% are infected through homosexual transmission, the major epidemic pattern of AIDS in China at present. Therefore, we chose Zhejiang and used the APC model to analyze the temporal trend of AIDS incidence in Zhejiang. Specifically, we assessed the age-period-cohort effect on AIDS incidence between 2004 and 2018 based on the whole population (including the local population and immigrant population) in Zhejiang, and studied the heterogeneity of AIDS incidence. In addition, we explored the characteristics of three transmission routes (homosexual, heterosexual transmission, injecting drug users and others) in these population groups.

## Methods

### Data source

The case data of this study were from the national Comprehensive AIDS Prevention and Control Information System - Antiviral Treatment Management database. We reviewed and downloaded the data of HIV/AIDS cases (including sex, date of birth, confirmed date, route of infection, current address, household registration and other information) reported in Zhejiang province from 2004 to 2018. Baseline population data included two data sources: age composition of the permanent residents in Zhejiang province (i.e., 0, 1, 2... 8, 9, 10 to 14, 15 to 19..., 80–84 years old, 85–100 years old) between 2004 and 2018 from the Chinese disease prevention and control information system; the annual local population data between 2004 and 2017 from the *Zhejiang Statistical Yearbook*. In this study, the migrant population refers to the people who flow into the city from other places, work and live in the city, but do not have the registered permanent residence of the city. The whole population is composed of local born population and migrant population. Therefore, the migrant population was calculated by subtracting the local population from the whole population (Additional file 1: Appendix A1). Due to the lack of data from the *Zhejiang Statistical Yearbook (2018)*, we were unable to obtain the annual local population data in 2018. Therefore, the AIDS case data of 2018 were excluded from the further analysis on local and immigrant populations.

### Statistical analysis

Incidence and mortality reflect the prevalence and death risk of the target population, as well as the health risk throughout their life [14, 15]. The incidence and mortality could not be estimated using the commonly-used statistical methods, so we used APC model based on Poisson distribution to explore the incidence or mortality of cancer and chronic disease, to explore the impact of age, period, and cohort on the incidence and mortality of the disease.

In order to facilitate the subsequent analysis and research, we processed the data of AIDS cases in the whole, local and immigrant populations as follows: (1) For AIDS cases in the whole and local populations, the first 15 age groups (groups aging 0–14 years) were clustered because the overall number of cases under 15 years of age was less than 0.1%. Since the overall number of AIDS cases over the age of 70 was less than 3%, we grouped the last 30 age groups (groups aging 71–100 years) together. We combined the whole population data (2004–2018) and local population data (2004–2017) with above two AIDS age groups respectively and divided them into 55 age groups. (2) For the AIDS cases in the immigrant population, since the number of AIDS cases between 0 and 15 years old was less than 0.1%, the first 15 age groups (group aging 0–14 years) were clustered as the group under 15 years old; since the number of AIDS cases over 50 years of age was less than 3%, the last 50 age groups (group aging 51–100 years) were clustered as one group. We combined data of immigrant population with immigrant AIDS cases from 2008 to 2017, and classified them into 35 age groups.

According to Willekens (1993) [16], logistic regression was used to fit APC model: *μ*_*xtk*_ = *e* ⋅ *a*_*x*_ ⋅ *b*_*t*_ ⋅ *c*_*k*_, where *μ*_*xtk*_ was a component of system, referring to influences of the *X* age group, the *t* cycle, and the *k* cohort (*set*{*x*,   *t*,   *k*}). *a*_*x*_ was the parameter of age *x*^*th*^, *b*_*t*_ was the parameter of period *t*^*th*^, *c*_*k*_ was the parameter of cohort *k*^*th*^. Adding natural logarithms on both sides of the equation, we got a logarithmic linear regression function: *y*_*xtk*_ = *Inμ*_*xtk*_ = *Ine* + *Ina*_*x*_ + *Inb*_*t*_ + *Inc*_*k*_. After converting, we got: *y*_*xtk*_ = *β*_1_*x*_*xtk*1_ + *β*_2_*x*_*xtk*2_ + *β*_3_*x*_*xtk*3_ + *β*_4_*x*_*xtk*4_, among which *x*_*xtkj*_ was a pseudo-variable, if *j*^*th*^ beta parameter was related to the age-period-queue category, and *x*^*xtkj*^ was equal to 1. We improved the function ${y}_{xtk}= In{\mu}_{xtk}=\sum \limits_{j=1}^N{\beta}_j{x}_{xtk,j}$, where *μ*_*xtk*_ was the system composition, referring to the age group *X*, the period *t*, and the influence of the cohort *k*(*set*{*x*,   *t*,   *k*}), *β*_*j*_ was the effect of *N* unknown parameters (currently three), *x*_*xtkj*, *j*_ was the pseudo variable of set {*x*,  *t*,  *k*} and *j*.

The prediction of the APC model requires the extrapolation of estimated parameters, which must be done without introducing identification problems [17, 18]. For different submodels, there are many different extrapolation possibilities, and the result of extrapolation is point predictions, on which distribution predictions should be built [19]. Therefore, we made linear predictions for all models, with distributed predictions provided by the Poisson response model and the over-scattered Poisson response model.

We analyzed the AIDS patients in single age groups using APC model based on Poisson distribution. Then APC analysis was performed for the annual incidence of AIDS transmitted through homosexual, heterosexual, and other routes (such as blood transfusion and intravenous drug abuse) in single age groups. In terms of minimizing bias and Akaike (AIC) information criteria [20, 21], the best model was selected. We bootstraped the sample data in the whole data set 100 times, fitted each sample with APC model to check the sensitivity and fitting performance of the final APC model, and drew a probability plot (Additional file 1: Appendix A2-A3). In general, all the models achieved good fitting.

The APC model was supposed to estimate the second order difference due to the existence of linear correlation among age, period, and cohort [22]. Logarithmic likelihood ratio tests were performed on the full and nested models of age-period, age-cohort, and period-cohort to test the statistical significance of age, period, and cohort effects. The t test was used to evaluate the difference of age effect among three groups. The smoothing spline was used to fit the future population based on sex [23] and AIDS cases transmitted through various routes. The incidence of AIDS in the next 5 years was estimated by applying the vector autoregression (VAR) [24] to the APC model.

All analyses, data visualization, and models were programmed in R (version 3.6.0). R package “apc” was used to fit the apc model and predict future trends [25].

## Results

### Descriptive analysis on AIDS incidence

Table 1 summarized the data on the incidence of AIDS transmitted through different routes in local and immigrant populations. Over these 15 years, a total of 27,308 AIDS cases were confirmed. Among them, there were 22,467 male cases and 4841 female cases. For the population groups, there were 18,287 cases in local population and 9023 in immigrant population. There were 15,050 cases of heterosexual transmission, 11,057 cases of homosexual transmission, and 1201cases of other transmission routes. In males, the number of heterosexually transmitted cases ranked the highest in the local population, while for immigrant, homosexual transmitted cases ranked the highest. The overall AIDS incidence was higher in males than in females. In local population, AIDS cases over 50 years old with heterosexual transmission accounted for the highest proportion, while the cases of homosexual transmission were mostly between 21 and 35 years old. In immigrant population, AIDS cases were mainly aged 21–35 years old. In both local and immigrant populations, the number of married/cohabiting patients transmitted through heterosexuality ranked the highest, while in unmarried patients, homosexual transmission was the highest. Most AIDS patients were at clinical stage I, and the number of cases was gradually reduced with the development of clinical stage.

**Table 1:** AIDS cases transmitted via various routes in local and immigrant populations

Variables Groups		Heterosexual transmission		Homosexual transmission		Others	
		(***N*** = 15,050)		(***N*** = 11,057)		(***N*** = 1201)	
		no.	%(95%CI)	no.	%(95%CI)	no.	%(95%CI)
**Sex**							
Local	Male	7927	52.67 (51.87–53.47)	6795	61.45 (60.54–62.36)	584	48.63 (45.76–51.49)
	Female	2812	18.68 (18.06–19.32)	5	0.05 (0.01–0.11)	162	13.49 (11.61–15.56)
Immigrant	Male	2582	17.16 (16.56–17.77)	4252	38.46 (37.55–39.37)	327	27.23 (24.73–29.84)
	Female	1729	11.49 (10.98–12.01)	5	0.05 (0.01–0.11)	128	10.66 (8.97–12.54)
**Age groups**							
Local	0–20	202	1.34 (1.16–1.54)	552	4.99 (4.59–5.41)	30	2.50 (1.69–3.55)
	21–35	2643	17.56 (16.96–18.18)	3853	34.85 (33.96–35.74)	245	20.40 (18.15–22.79)
	36–50	3346	22.23 (21.57–22.91)	1681	15.20 (14.54–15.89)	253	21.07 (18.79–23.48)
	Over50	4548	30.22 (29.49–30.96)	714	6.46 (6.01–6.93)	218	18.15 (16.01–20.45)
Immigrant	0–20	195	1.30 (1.12–1.49)	251	2.27 (2.00–2.57)	23	1.92 (1.22–2.86)
	21–35	2190	14.55 (13.99–15.13)	2796	25.29 (24.48–26.11)	242	20.15 (17.91–22.53)
	36–50	1532	10.18 (9.70–10.67)	1094	9.89 (9.34–10.47)	163	13.57 (11.68–15.64)
	Over50	394	2.62 (2.37–2.89)	116	1.05 (0.87–1.26)	27	2.25 (1.49–3.25)
**Marital status**							
Local	Unmarried	1768	11.75 (11.24–12.27)	4243	38.37 (37.47–39.29)	186	15.49 (13.49–17.66)
	Married/Cohabiting	6888	45.77 (44.97–46.57)	1739	15.73 (15.05–16.42)	430	35.80 (33.09–38.59)
	Divorced/Separated	1608	10.68 (10.20–11.19)	768	6.95 (6.48–7.44)	94 7.83 (6.37–9.49)	
	Widowed	460	3.06 (2.79–3.34)	39	0.35 (0.25–0.48)	21	1.75 (1.09–2.66)
	Unknown	18	0.12 (0.07–0.19)	11	0.10 (0.05–0.18)	15	1.25 (0.70–2.05)
Immigrant	Unmarried	1152	7.65 (7.23–8.09)	2713	24.54 (23.74–25.35)	147	12.24 (10.44–14.23)
	Married/Cohabiting	2390	15.88 (15.30–16.47)	987	8.93 (8.40–9.47)	236	19.65 (17.44–22.01)
	Divorced/Separated	648	4.31 (3.99–4.64)	542	4.90 (4.51–5.32)	53	4.41 (3.32–5.73)
	Widowed	110	0.73 (0.60–0.88)	11	0.10 (0.05–0.18)	10	0.83 (0.40–1.53)
	Unknown	11	0.07 (0.04–0.13)	4	0.04 (0.01–0.09)	9	0.75 (0.34–1.42)
**Clinical stage**							
Local	Clinical phase I	6347	42.17 (41.38–42.97)	4187	37.87 (36.96–38.78)	512	42.63 (39.81–45.48)
	Clinical phase II	1964	13.05 (12.52–13.60)	1534	13.87 (13.23–14.53)	101	8.41 (6.90–10.12)
	Clinical phase III	1492	9.91 (9.44–10.40)	699	6.32 (5.88–6.79)	87	7.24 (5.84–8.86)
	Clinical phase IV	936	6.22 (5.84–6.62)	380	3.43 (3.10–3.79)	46	3.83 (2.82–5.08)
Immigrant	Clinical phase I	2861	19.01 (18.39–19.65)	2560	23.15 (22.37–23.95)	312	25.98 (23.52–28.56)
	Clinical phase II	899	5.97 (5.60–6.36)	1185	10.72 (10.15–11.31)	94	7.83 (6.37–9.49)
	Clinical phase III	352	2.34 (2.10–2.59)	345	3.12 (2.80–3.46)	35	2.91 (2.04–4.03)
	Clinical phase IV	199	1.32 (1.15–1.52)	167	1.51 (1.29–1.76)	14	1.17 (0.64–1.95)

**Fig. 1:**
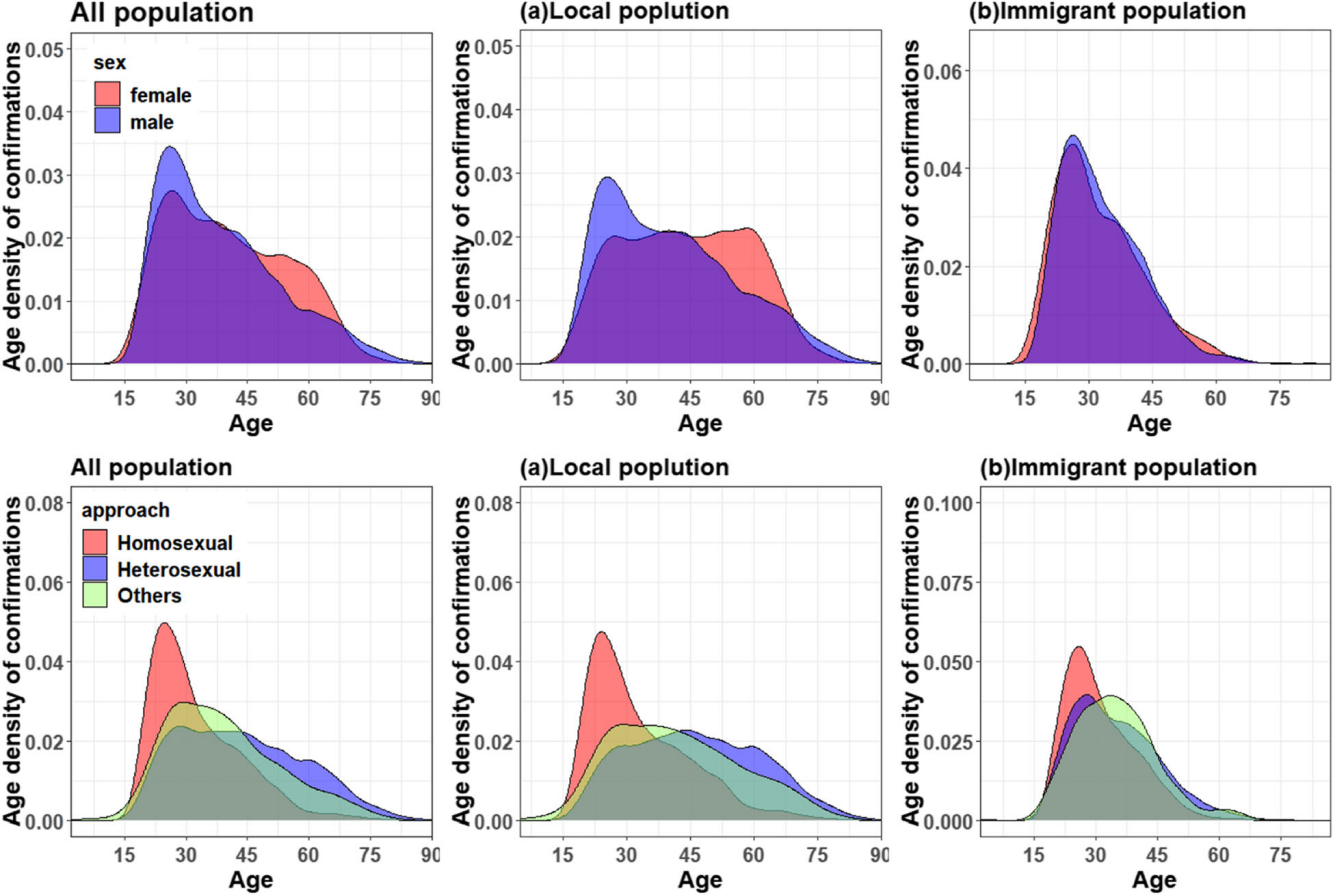
The AIDS age density in males and females, and age density of AIDS via different transmission routes in the whole population (including **a**. local population and **b**. immigrant population). In the diagrams above, blue, and red represent the age density of males and females, respectively. In the diagrams below, red, blue, and green represent the age density of AIDS via homosexual, heterosexual, and other transmission routes, respectively

In Fig. 1, we depicted the age density of AIDS incidence based on different sexes, as well as age density of AIDS incidence based on different transmission routes in the whole, local and immigrant populations. The characteristics of AIDS incidence were as follows. First, significant differences in age density were found between males and females, and the density of AIDS incidence in males of 20–35 years old was higher than in females of the same age. Females of 45–65 years old had large-scale infection, and the age density of AIDS incidence in females was higher than in males. In immigrant population, the age density of AIDS incidence in males and females was similar, and the large-scale infection all occurred to those between 20 and 35 years old. The age density of AIDS cases via homosexual transmission showed cluster phenomenon and peaked between 20 and 35 years old. AIDS cases via heterosexual transmission and other routes (blood transfusion, intravenous drug abuse) showed large-scale infection between 20 and 45 years old.

**Fig. 2:**
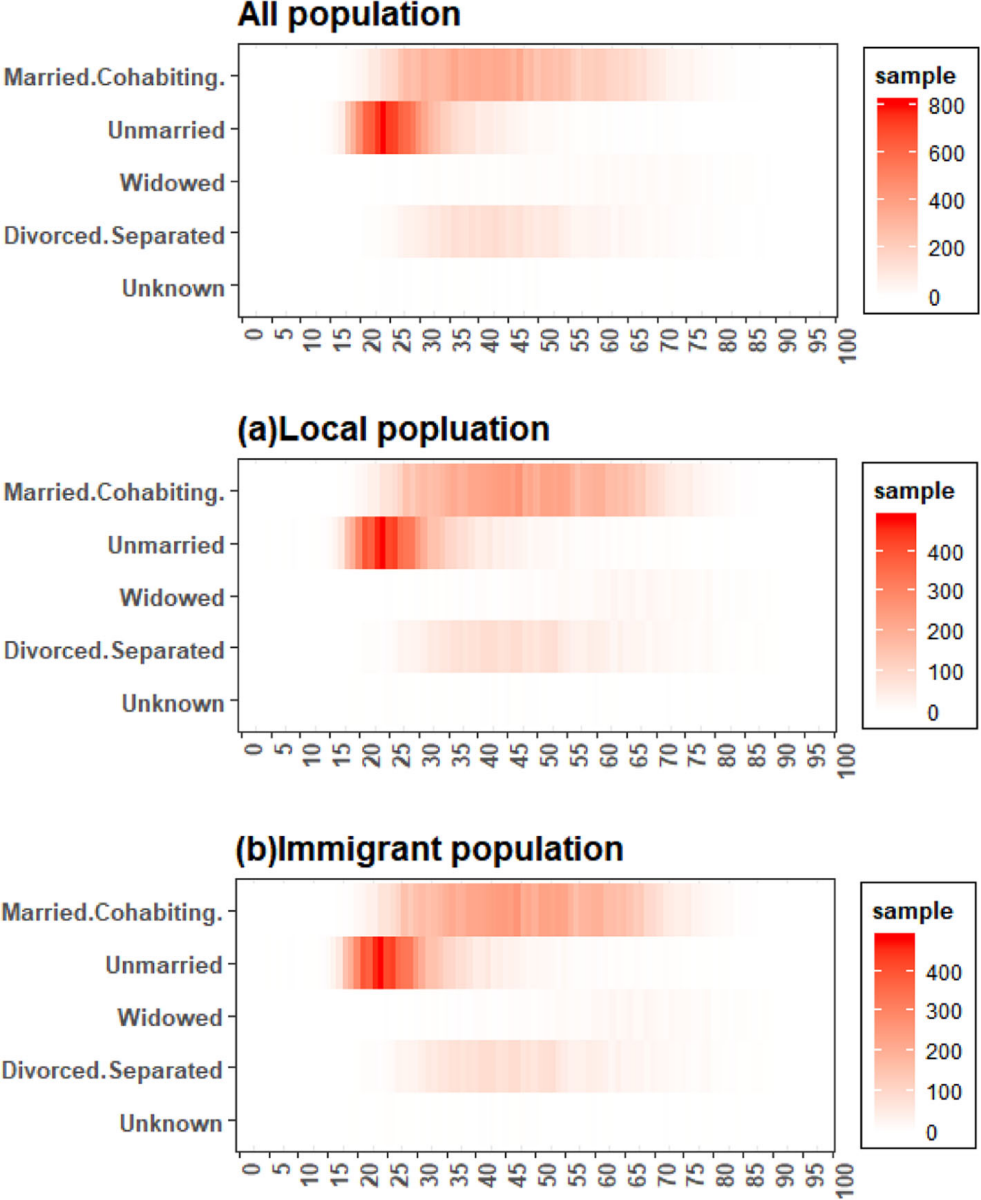
Age distribution of AIDS cases with different marital status in Zhejiang, China

Figure 2 elucidated the age distribution of AIDS cases with various marital status in Zhejiang, China. The number of AIDS patients was the highest in the unmarried, and married or cohabiting groups, followed by divorced or separated groups. The unmarried AIDS cases were mostly between 15 and 35 years old, the married or cohabiting AIDS cases were mostly between 25 and 70 years old, and divorced or separated AIDS cases were mostly between 30 and 55 years old.

### APC analysis on incidence of AIDS in different populations

**Fig. 3:**
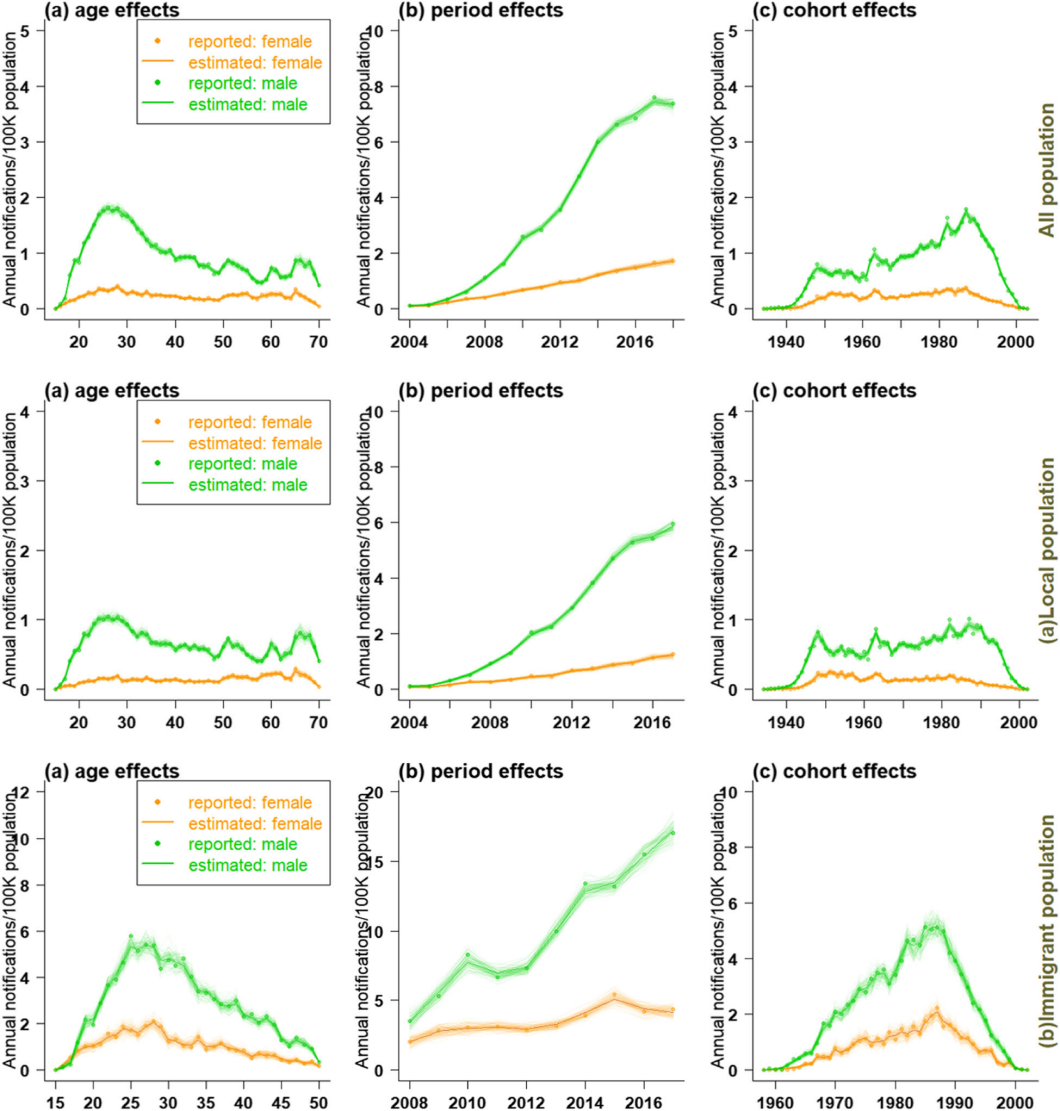
The effect of age (left), period ((middle), and cohort (right) on the estimation of AIDS incidence in all populations (**a**: local population, **b**: immigrant population)

**Fig. 4:**
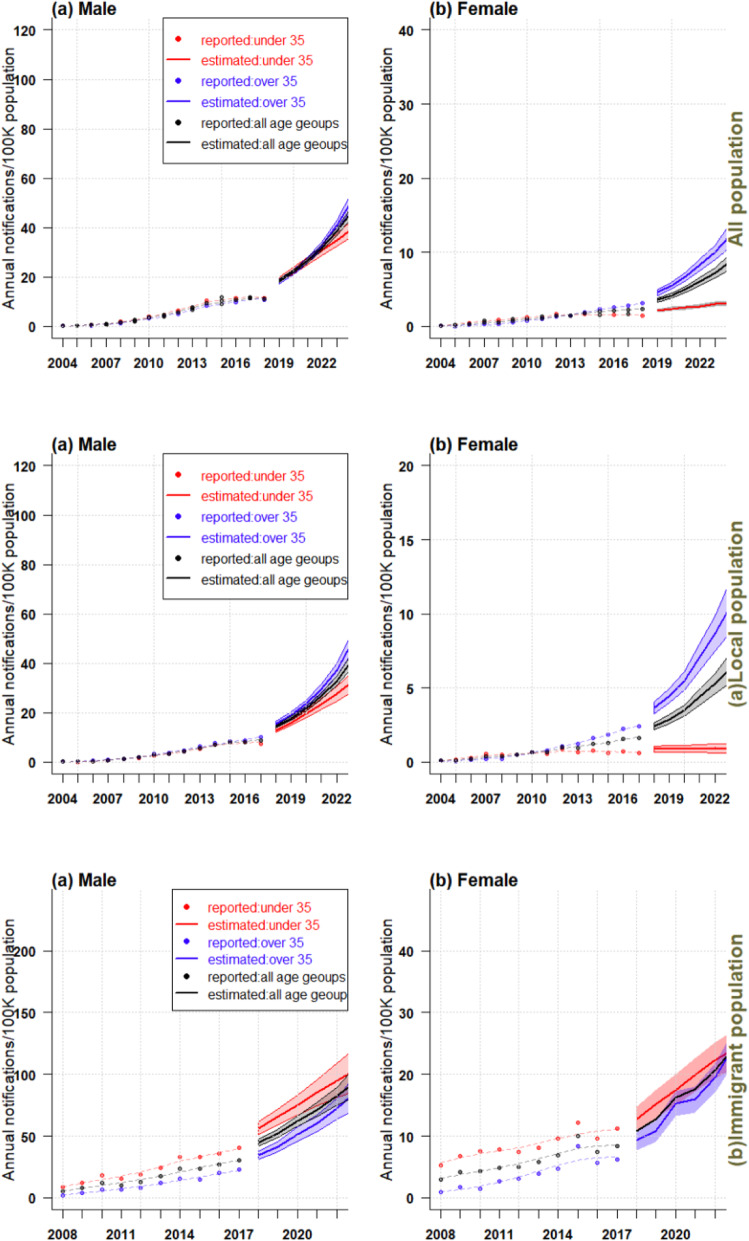
Trends of AIDS incidence and projected trends over the next 5 years in the whole population of Zhejiang, China (**a**. local population, **b** immigrant population)

Figures 3 and 4 showed the effect of age, period, and cohort on the estimation of AIDS incidence and the predicted trend in different populations. Due to the gender difference in the age density of AIDS onset, we selected suitable APC model for men and women in different populations by minimizing bias and Akaike information criterion (AIC) (See Additional file 1: Appendix A4: tables 2–7).

### Age effect

In the whole population, as well as the local and immigrant populations, the AIDS incidence in males peaked between 20 and 35 years old. In all population and the local population, the AIDS incidence in males showed a relatively stable trend after 35 years old and peaked between 60 and 70 years old again. In immigrant population, the incidence of AIDS in males aged over 35 decreased sharply. For females, the age effect on AIDS incidence was relatively lower than in males. In whole and local females, AIDS incidence showed a slowly increasing trend between 20 and 35 years old; it remained stable after 35 years old and gradually decreased over 65 years old. In immigrant females, the incidence of AIDS peaked between 20 and 35 years old, and then decreased slowly. Based on the above results, the effect of age on AIDS incidence could be divided into two stages: over 35 years old and below 35 years old.

### Period effect

Influenced by period, the AIDS incidence in males was higher, and increased at a faster speed than females. In the whole and local populations, the incidence in males rose sharply after 2005, reaching to 6–8 cases per 100,000 population during the study period. The incidence in females rose slowly from 2005 to 2018 (remaining at around 1–2 cases per 100,000 population). Among the immigrant population, the AIDS incidence of males rose sharply from 2008 to 2010, then leveled off between 2010 and 2012 (7–8 cases per 100,000 population), and rose sharply between 2012 and 2017 (16–17 cases per 100,000 population). The AIDS incidence in females leveled off throughout the study period, maintaining at 3–5 cases per 100,000 population.

### Cohort effect

The cohort effect showed a similar pattern for the whole and local populations, with the incidence of AIDS peaking in males born in 1950, 1965 and 1985–1990, and falling sharply in those born after 1990. The cohort effect exerted little influence on the incidence of AIDS among females. There was a small peak in AIDS incidence among the females born between 1985 and 1990 in the whole population, and the incidence declined slowly thereafter. In local population, the incidence of AIDS peaked in females born around 1950. The incidence of AIDS among immigrant population was significantly affected by the cohort effect, reaching a peak in both males and females born between 1985 and 1990, and declining sharply thereafter.

### Prediction of AIDS incidence in different age groups in the whole, local and immigrant populations

The AIDS incidence showed an upward trend from 2004 to 2008 in males over 35 years old, below 35 years old and of all age groups, and in females over 35 years old and of all age groups; it was also estimated that a sharply increasing trend would occur in the 5 years to come, and the final incidence in people over 35 years old would be higher than those below 35 (Fig. 4). In the whole population, the AIDS incidence of females under 35 years old showed no obvious changes between 2004 and 2018, however, it was predicted to increase in the future. In the local population, the incidence among females under 35 years old remained stable (1 case per 10,000 population). In the immigrant population, the incidence of both males and females below the age of 35, over 35 and of all age groups showed a significant upward trend during the study period (2008–2017). The incidence of both males and females below 35 was higher than those over 35, and this trend was expected to continue in the next 5 years.

### APC analysis on the incidence of AIDS via various transmission routes in different populations

**Fig. 5:**
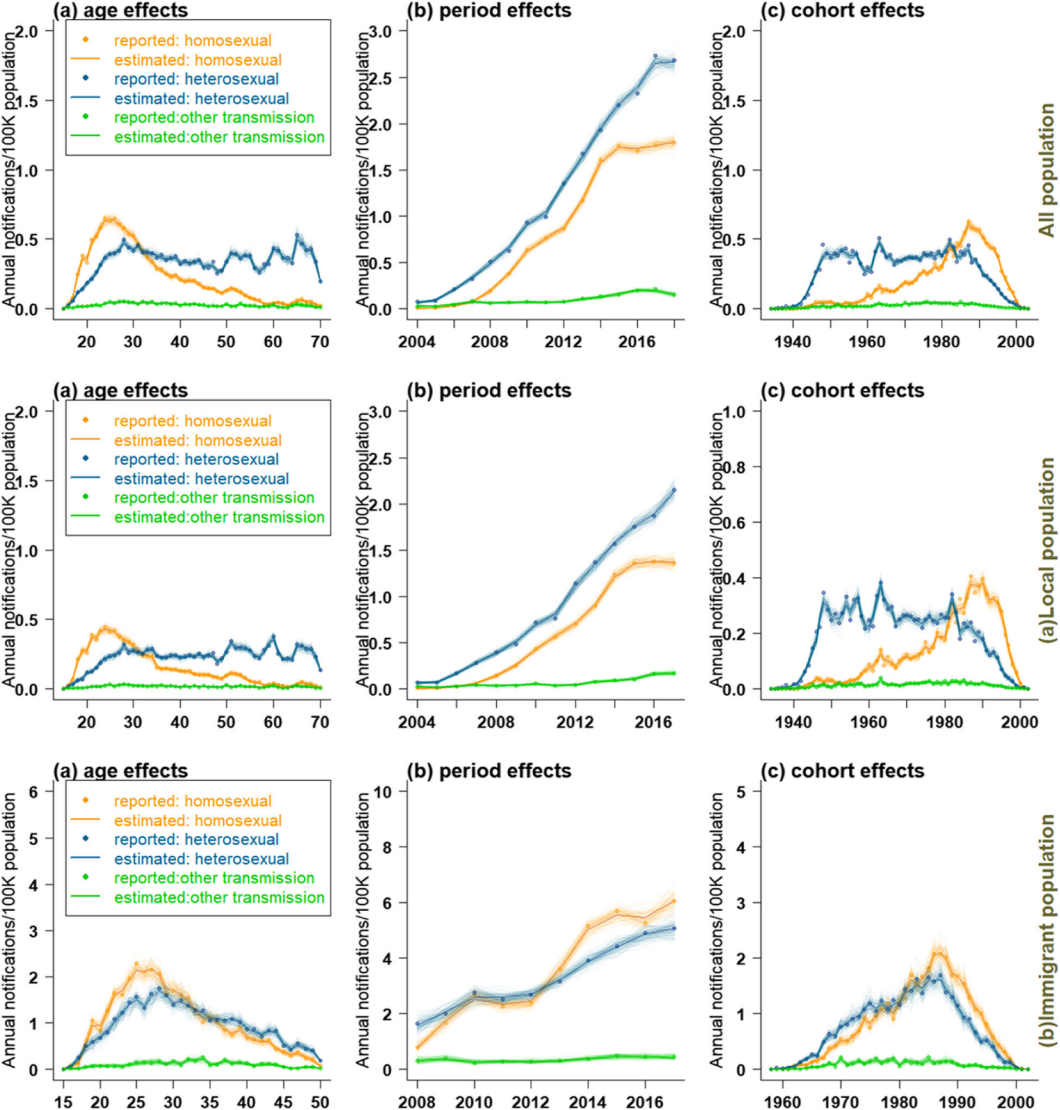
Estimated impact of age (left column), period (middle column) and birth cohort (right column) on AIDS incidence via different transmission routes in the whole population (including **a**. local population and **b**. immigrant population) of Zhejiang Province, China

**Fig. 6:**
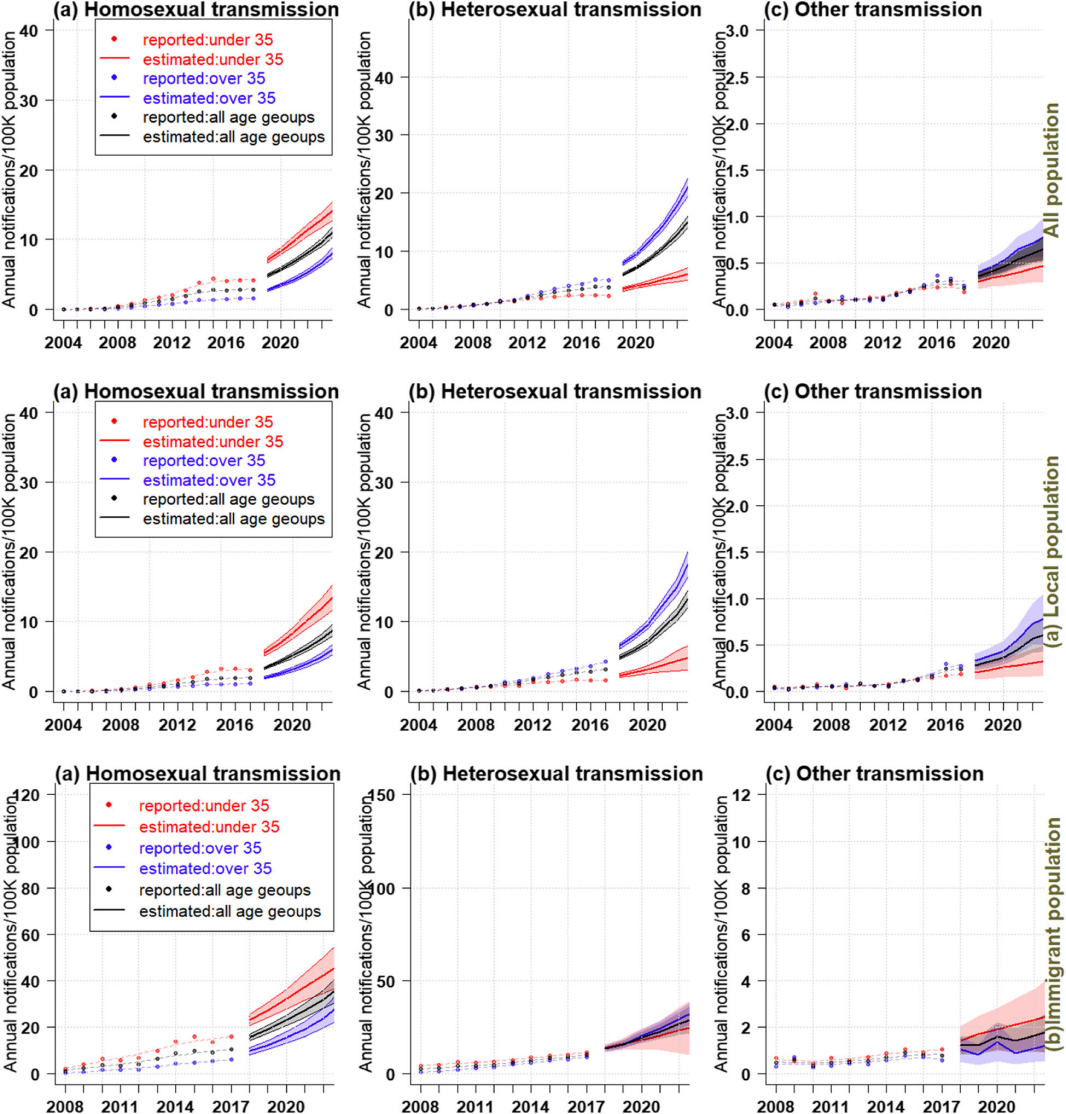
Trends in AIDS incidence via different transmission routes in Zhejiang Province, China (including **a**. local and **b**. immigrant populations), and projected trends over the next 5 years

Figures 5 and 6 showed the estimated impact of age, period, and birth cohort of the whole population, including the local and immigrant populations in Zhejiang, on the incidence of AIDS through different transmission routes, and the projected trends. Considering that the age density of AIDS patients with different transmission routes varied (Fig. 1), APC models were also established for each transmission routes. Results of APC analysis of AIDS cases through various transmitted routes in different population groups has been shown in Additional file 1: Appendix A5 ( tables 8–6).

### Age effect

In both the whole and the local populations, the incidence of AIDS through homosexual transmission peaked significantly between 20 and 35 years old, and declined sharply after 35 years of age. The annual age-specific incidence of AIDS via homosexual transmission rose sharply before the age of 30, followed by fluctuations, but the overall trend leveled off. The incidence of AIDS via homosexual transmission was significantly higher than that of heterosexual before 31 years of age, while after 31 years of age, the incidence of AIDS via heterosexual transmission was significantly higher than that of homosexual. Among the immigrant population, a similar pattern of age-related effects was observed in both homosexual and heterosexual AIDS patients, with annual age-specific incidence peaking between 25 and 30 years of age, then falling sharply. The incidence of homosexual transmission was higher than heterosexual transmission before 35 years old and higher than that of homosexual transmission after 35. The age effect had little impact on the incidence of AIDS transmitted via other routes in the whole, local, or immigrant populations.

### Period effect

Under the influence of the period effect, the incidence of homosexual transmission in the whole and the local populations increased till 2015 and then remained stable (about 2 cases per 100,000 people). Throughout the whole study period, the incidence of AIDS via heterosexual transmission rose sharply to about 2–3 per 100,000 people, and remained higher than that via homosexual transmission. In general, the incidence of HIV/AIDS through homosexual and heterosexual transmission in the immigrant population was on the rise. From 2012 to 2016, the incidence of HIV/AIDS transmitted homosexually and heterosexually had increased rapidly, from 2 to 5–6 cases per 100,000 people. Before 2012–2013, the incidence of AIDS via transsexual transmission was higher than that via heterosexual transmission, and then turned lower after 2013. The period effect had little effect on the incidence of AIDS transmitted by other routes in the total, local and immigrant populations.

### Cohort effect

In the whole and local populations, the incidence of AIDS via homosexual transmission peaked among those born in 1990. Significantly affected by the cohort effect, the incidence of AIDS via heterosexual transmission rose sharply among those born between 1940 and 1950, then fluctuated until a sharp decline among those born around 1985. In patients born before 1985, the incidence of AIDS via heterosexual transmission was significantly higher than that via homosexual transmission. And for those born after 1985, the incidence of AIDS via heterosexual transmission was significantly lower than that via homosexual transmission. In the immigrant population, the cohort effect exerted a significant impact on the incidence of AIDS via transsexual and heterosexual transmission among those born between 1985 and 1990. The cohort effect had little impact on the incidence of AIDS transmitted by other routes, whether in the total, local, or immigrant populations.

### Prediction of AIDS in various age groups among different populations in future 5 years

Figure 6 showed the incidence trends of AIDS via different transmission routes among the whole, local and immigrant populations, and the predicted trends over the next 5 years. Overall, the prevalence of AIDS, whether transmitted by homosexual, heterosexual, or other routes, would be increasing over the next 5 years in all three age groups. The characteristics of the estimated incidence were as follows: (1) The incidence of AIDS via homosexual transmission in those below 35 years of age was nearly twice as high as in those over 35 in the whole, local and immigrant populations; (2) The incidence of AIDS via heterosexual transmission in those over 35 was significantly higher than those below 35 in the whole and immigrant populations; (3) For other modes of transmission, the incidence of AIDS in cases over 35 years old was higher than those below 35 years old in the whole and local populations. In the immigrant population, the AIDS incidence of people over 35 was lower than those below 35.

## Discussion

APC model, which is usually used for chronic diseases [26–28], can be applied to infectious diseases such as hepatitis B, tuberculosis, and AIDS [29, 30], as these are all long-term infections. Therefore, we used APC model to analyze the characteristics of HIV/AIDS incidence in both males and females in the whole, local and immigrant populations in Zhejiang, and further explored the incidence trend among HIV/AIDS patients infected by different transmission routes.

Age is an important influencing factor of AIDS. Our results showed that the incidence of AIDS was significantly affected by age in both males and females between 20 and 35 years old in the whole, local and immigrant populations, suggesting a higher AIDS risk among young people. This might be attributed to unprotected sex and multiple sexual partners [31]. A large part of people in this age group were college students, and many of them were immigrants. According to data from The Chinese Center for Disease Control and Prevention (CDC), in the past few years, the number of college students newly diagnosed with AIDS increased by 30 to 50% annually [32]. Our study also elucidated that people with sexually transmitted infections (especially homosexual transmission) tended to be younger [33]. Homosexual transmission has become a major way of AIDS infection among college students. Unfortunately, many homosexual male students know nothing about AIDS until they are tested positive for HIV infection [34]. Therefore, it is urgent to promote health education about sex and raise the awareness of AIDS prevention among college students. The second peak of HIV/AIDS in the whole and local populations appeared at the age of 60–70, which can be attributed to the ageing population in China [35]. The elderly are more susceptible to diseases and injuries [36, 37]. Some studies have shown that [38] the proportion of elderly people suffering from AIDS is much higher than before. The impact of the age effect on young immigrants of 20–35 years old can also be explained by the fact that immigrants are mostly engaged in physically demanding jobs and poor housing conditions, which limit their access to health care and health information, and thus increase their risk of HIV infection [39].

The policy of reform and opening-up of China has promoted population mobility, and this also has an impact on the HIV epidemic in a variety of interrelated ways [40]. In our study, there is a significant difference in the incidence of AIDS among the whole population and the local born population, and the immigrant population under the influence of age effect. The male AIDS patients in the immigrant population aged 35–50 were significantly affected by age effect, while the male AIDS patients in the whole population and the local born population were not. This may be due to the increased number of migrant workers aged 35–50 with the rapid economic development in recent years [41]. Among the immigrants, the age effect was significant in those between 15 and 50 years old, while in the whole population and the local born population, people aged between 20 and 35 years old were influenced obviously. The age effect was significant in those between 20 and 70 years old with heterosexual transmission as well. Studies have shown that young who have sex with men (MSM) often use homosexual mobile apps, and therefore, compared with older MSM, they have a broader and more dynamic partnership network [42]. This may explain why AIDS prevalence is increasing among 20- to 35-year-old MSM.

The period effect showed that the incidence of HIV/AIDS in males was consistently higher than that in females during the study period, and the rate of increase in males was significantly higher than that in females, which conforms to the results of other studies [43, 44]. The incidence among immigrant males was the highest, rising sharply from 2008 to 2010, then leveling off from 2010 to 2012 (7–8 per 100,000 population) and rising sharply again from 2012 to 2017 (16–17 per 100,000 population). The number of HIV/AIDS cases via homosexual and heterosexual transmission in the immigrant population continued to rise from 2008. This can be explained as follows: On one hand, due to social discrimination, a considerable proportion of MSM leave their registered area to work and live in other areas, thus having a high mobility [45]. The mobility of HIV-infected MSM often leads to an increase of epidemic in the destination area and even second-generation transmission. Qin et al. [46] showed that from 2008 to 2015, the number of migrant HIV infection cases of by male and male sex behavior in China increased, and the proportion of migrant, young, unmarried patients with a high education level and household registration (hukou) in rural areas was relatively high. On the other hand, heterosexual transmission is still a major transmission route of AIDS epidemic in Zhejiang Province. In addition, migrant workers form a high-risk group of HIV infection. Immigrants from rural to urban areas are at a high risk of HIV infection [47], but they lack the knowledge about HIV. Yang et al. [48] showed that about 40% immigrants did not know that the use of condoms can reduce the risk of HIV infection. Due to the current hukou system in China, it is difficult for immigrants to obtain hukou in the destination city, especially in economically developed regions like Zhejiang. And in the destination cities, health services for immigrants remain underutilized and unevenly distributed. The frequently changing government regulations on health services and prevention planning for migrant population have increased uncertainty in policy implementation and exposed migrant populations to the risk of HIV transmission [49]. A survey using national demographic data has showed that areas with a higher HIV prevalence have higher rates of HIV-related education among migration population; compared with the central and western regions in China, in the eastern coastal areas with more medical and financial resources, the proportion of migrant population who have received HIV education is relatively low [50]. Hence, health care resources need to be allocated to areas with large migrant populations so as to provide better health care for migrant workers.

Cohort effects reflect changes in early life environments and assume that people in the same birth cohort have equal exposure to the risk factors of a disease. Exposure to adverse environmental factors early in life may exert adverse effects on later life [51]. In the entire study cohort, the incidence of AIDS peaked in males born around 1990 and declined sharply in males born after 1990, while the incidence of AIDS in females was less affected by the cohort effect in the whole population as well as the local and immigrant populations. Furthermore, the incidence of HIV transmitted by homosexual and heterosexual sex was significantly influenced by cohort effects between 1985 and 1990, which is speculated to be related to a major epidemic among commercial plasma donors in the central and eastern provinces around 1990 [2], but further research is needed.

The age density diagrams of HIV/AIDS onset and transmission routes in both sexes (Fig. 1) showed widespread HIV infections in males and females between the ages of 20 and 35; the same was found in the immigrant population. Homosexual transmission of HIV/AIDS cases surged in those between 20 and 35 years old. AIDS cases by heterosexual transmission, blood transfusion, intravenous drug use, and other ways of transmission concentrated in population between 20 and 45 years old. As shown in Fig. 2, there was a relatively large number of unmarried AIDS patients aged 20–30. Therefore, more attention should be paid to the HIV infection of unmarried population of this age group and appropriate AIDS control programs need to be developed.

Our predictive results showed that in the whole and local populations, the AIDS incidence in males and females of all age groups would rise sharply in the next 5 years; in 2023, the AIDS incidence among the population over 35 years old would be significantly higher than those under 35 years old. The incidence in women under 35 years of age in the whole population was expected to increase slightly in the future. The trend remained the same for women under 35 years of age in the local population. Among the immigrant population, both males and females under 35 and over 35, as well as all age groups, were expected to have a significantly increased incidence in the next 5 years. The prevalence of HIV in all three groups of transmission routes would rise in the next 5 years. We therefore recommend that public health authorities in Zhejiang focus on the control of AIDS epidemic in the above-mentioned populations. Our findings also indicate the sex differences in AIDS incidence and suggest measures be devised specifically for males and females to control AIDS.

In this study, we analyzed the prevalence of HIV/AIDS in the local and immigrant populations of Zhejiang, a representative of economically developed regions, and further explored the characteristics of HIV/AIDS through different transmission routes. The main limitation of our study is the rough statistics of age distribution in the study populations, which may incur small noises and errors to the estimation. Therefore, more detailed statistics and census are needed to improve the accuracy of estimation and prediction.

## Conclusions

Our results suggest that HIV/AIDS exerts a significant impact on young people in different population groups, especially college students, and its incidence will continue to increase in the future. Due to unprotected sex, multiple sex partners, and lack of sex and AIDS prevention education, the burden of AIDS may still increase in Zhejiang in the next few years. The results of the study also indicate the sex difference in the incidence of AIDS, so measures to control AIDS incidence should be proposed specifically for males and females. It is hoped that public health departments in Zhejiang could focus on these issues so as to better control the AIDS epidemic.

## Supplementary Information

**Additional file 1.**

## Data Availability

Currently, the database used to support this study are not freely available inview of participants’privacy protection but are available from the corresponding author on reasonable data request. Researchers interested in our study could contact the corresponding author Dr. Zhihang Peng (zhihangpeng@njmu.edu.cn) who will review the data request.

## Abbreviations

AID: Sacquired immunedeficiency syndrome
APC: Age-Period-Cohort
HIV: Human immunodeficiency virus
AIC: Akaike information criteria
VAR: Vector autoregression
