# Supplementary material for: Analysis on the trend of AIDS incidence in Zhejiang, China based on the age-period-cohort model (2004–2018)
Source: BMC Public Health. 2021 Jun 5;21:1077. doi: 10.1186/s12889-021-11050-x (PMC8180133; doi:10.1186/s12889-021-11050-x)
Supplement: Supplementary file 1 — Additional file 1. [file 12889_2021_11050_MOESM1_ESM.docx]

**Appendix**

**A.1 data processing**

In order to calculate the exact populations of all ages, we used the following steps: (1) For the whole population, we subdivided the whole population into age groups, and calculated the local population of each age group based on the proportion of each age in the local population; (2) For the local population, we calculated the detailed age composition ratio of the permanent population, then combined the ratio with the data from Zhejiang local population statistical yearbook, and obtained the information on age structure of the local population. Then, according to the proportion of each age group, we calculated the population of each age group; (3) For the immigrant population, we combined the information on the resident population and that on the local population in the statistical yearbook to calculate the immigrant population of Zhejiang from 2004 to 2017, and then processed the data the same way as in (2).

**A.2 The fitted performance of age-period-cohort model for males and females of all populations (a. local population, b. immigrant population) divided by age-period in Zhejiang province**

The black spot represents the observed value that conforms to 80% quantile of Poisson distribution. The green triangle represents 5-10% (up) and 90-95% (down); The blue triangle represents 1-5% (up) and 95-99% (down); The red triangle represents the two ends of 1%. The closer the APC model fitting is to the median of a prior Poisson distribution (i.e., 50% percentile), the better the fitting performance is. Therefore, the higher the proportion of black spots is, the higher the goodness of fit is.

**a. The whole population (left: males, right: females)**

**
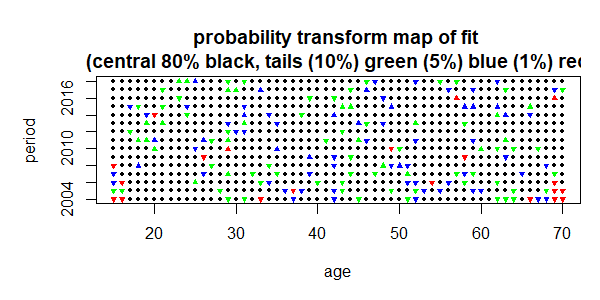

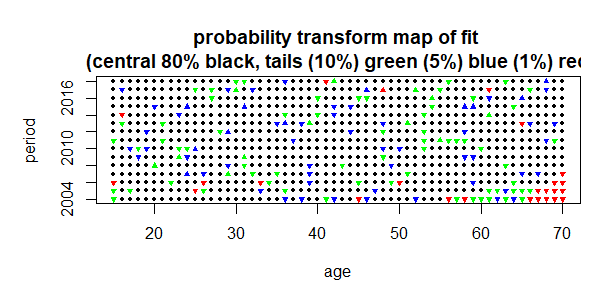
**

**b. The local population (left: males, right: females)**

**
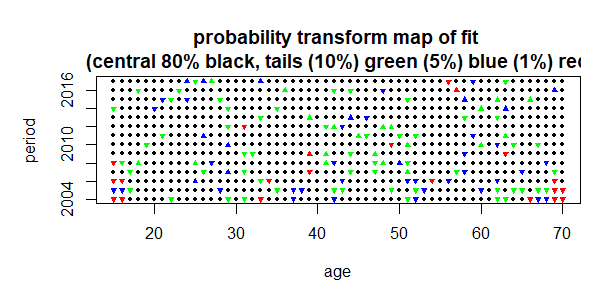

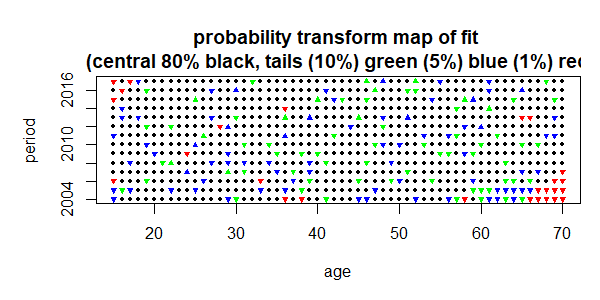
**

**c. The immigrant population (left: males, right: females)**

**
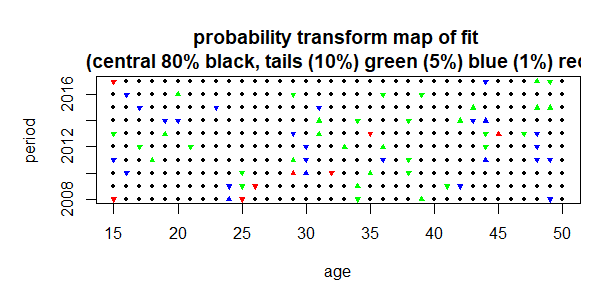

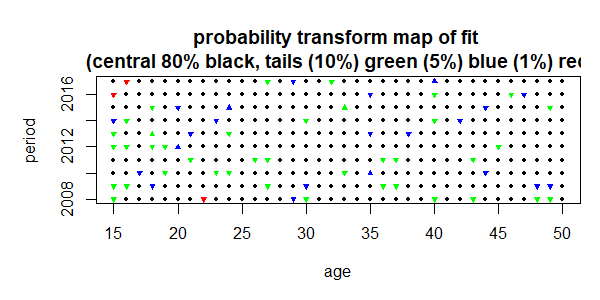
**

**A.3 The fitted performance of age-period-cohort model for all populations transmitted via different routes (a. local population, b. immigrant population)**

**a. The whole population (upper left: homosexual transmission; upper right: heterosexual transmission; down left: other transmission routes)**

**
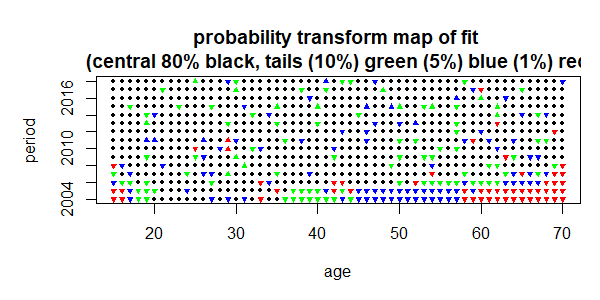

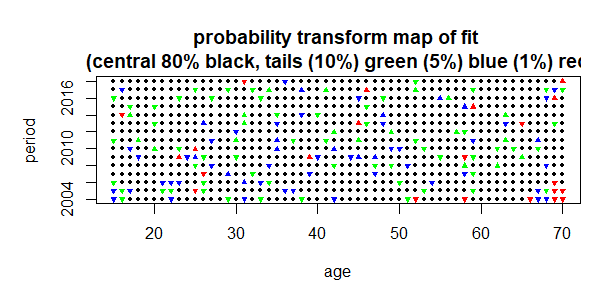
**

**
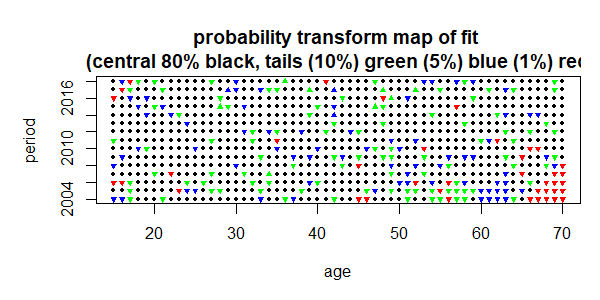
**

**b. The local population (upper left: homosexual transmission; upper right: heterosexual transmission, down left: other transmission routes)**

**
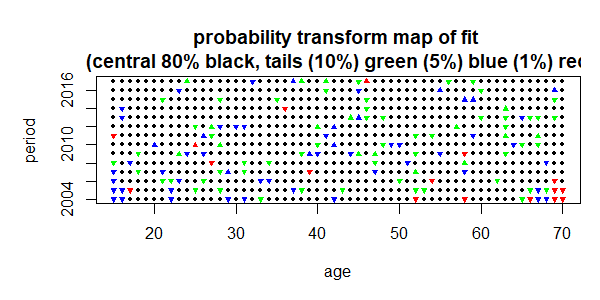

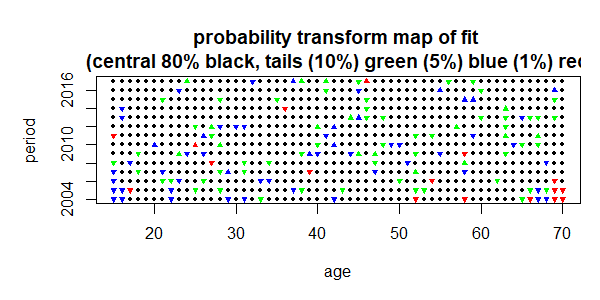
**

**
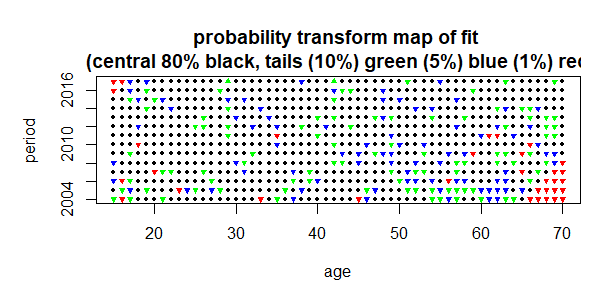
**

**c. The immigrant population (upper left: homosexual transmission; upper right: heterosexual transmission; down left: other transmission routes)**

**
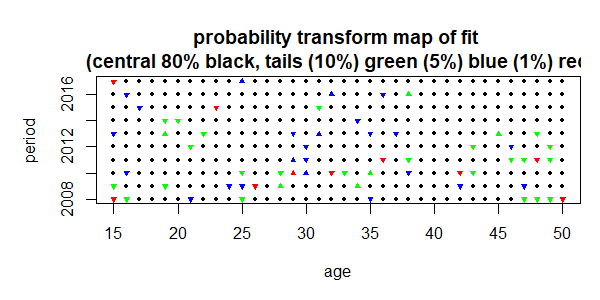

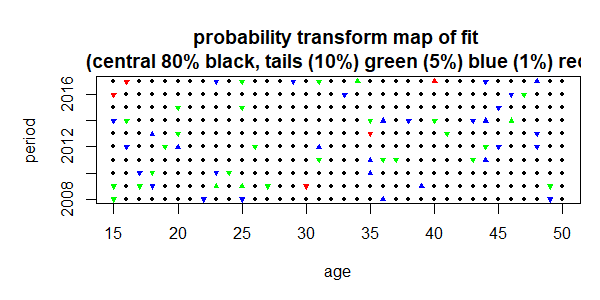
**

**
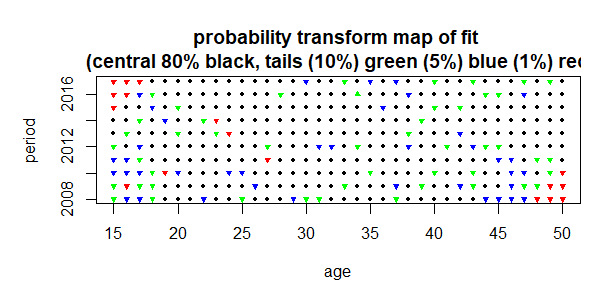
**

**A.4 APC analysis of AIDS cases of the whole, local, and immigrant populations based on different genders in Zhejiang,China**

**Table 2 Fitted APC results of man AIDS cases in the whole population**

| Model# | Increase of deviance | *p*-value of LR tests | Decrease in d.f. | AIC |
| --- | --- | --- | --- | --- |
| Full APC model | NA | NA | NA | 4512.076 |
| Sub-models |  |  |  |  |
| AP | 1360.847 | <0.001 | 68 | 4913.447 |
| AC | 2500.885 | <0.001 | 13 | 6163.485 |
| PC | 4737.358 | <0.001 | 54 | 8317.959 |
| Ad | 3059.392 | <0.001 | 81 | 6585.992 |
| Pd | 13285.91 | <0.001 | 122 | 16730.51 |
| Cd | 6517.757 | <0.001 | 67 | 10072.357 |
| A | 15692.653 | <0.001 | 82 | 19217.253 |
| P | 13286.299 | <0.001 | 123 | 16728.899 |
| C | 21972.853 | <0.001 | 68 | 25525.453 |
| t | 15107.964 | <0.001 | 135 | 18526.565 |
| tA | 28573.982 | <0.001 | 136 | 31990.582 |
| tP | 15108.449 | <0.001 | 136 | 18525.049 |
| tC | 28213.969 | <0.001 | 136 | 31630.569 |
| Constant only | 28620.29 | <0.001 | 137 | 32034.89 |

# The codes for the sub-models are as follows. AP: age-period model. AC: age-cohort model. PC: period-cohort model. Ad: age drift model. Pd: period drift model. Cd: cohort drift model. A: age model. P: period model. C: cohort model. t: trend model. tA: trend age model. tP: trend period model. tC: trend cohort model.

Abbreviations: LR: likelihood-ratio. d.f.: degree of freedom. AIC: Akaike information criterion.

**Table 3 Fitted APC results of woman AIDS cases in the whole population**

| Model^#^ | Increase of deviance | *p*-value of LR tests | Decrease in d.f. | AIC |
| --- | --- | --- | --- | --- |
| APC | NA | NA | NA | 3433.839 |
| Sub-models |  |  |  |  |
| AP | 1373.892 | <0.001 | 68 | 3903.499 |
| AC | 972.209 | <0.001 | 13 | 3611.816 |
| PC | 1810.844 | <0.001 | 54 | 4368.451 |
| Ad | 1525.62 | <0.001 | 81 | 4029.227 |
| Pd | 3631.971 | <0.001 | 122 | 6053.578 |
| Cd | 1973.606 | <0.001 | 67 | 4505.213 |
| A | 3282.035 | <0.001 | 82 | 5783.642 |
| P | 3672.563 | <0.001 | 123 | 6092.17 |
| C | 4099.527 | <0.001 | 68 | 6629.134 |
| t | 3798.151 | <0.001 | 135 | 6193.758 |
| tA | 5638.219 | <0.001 | 136 | 8031.826 |
| tP | 3839.517 | <0.001 | 136 | 6233.124 |
| tC | 5717.952 | <0.001 | 136 | 8111.559 |
| Constant only | 5719.387 | <0.001 | 137 | 8110.994 |

# the sub-model codes are the same as in Table 2.

Abbreviations: LR: likelihood-ratio. d.f.: degree of freedom. AIC: Akaike information criterion.

**Table 4 Fitted APC results of man AIDS cases in the local population**

| Model^#^ | Increase of deviance | *p*-value of LR tests | Decrease in d.f. | AIC |
| --- | --- | --- | --- | --- |
| Full APC model | 686.778 | 0.141 | NA | 3865.488 |
| Sub-models |  |  |  |  |
| AP | 1200.307 | <0.001 | 67 | 4245.017 |
| AC | 1523.23 | <0.001 | 12 | 4677.94 |
| PC | 2991.759 | <0.001 | 54 | 6062.469 |
| Ad | 1970.197 | <0.001 | 79 | 4990.907 |
| Pd | 6764.082 | <0.001 | 121 | 9700.792 |
| Cd | 3783.462 | <0.001 | 66 | 6830.172 |
| A | 10165.494 | <0.001 | 80 | 13184.204 |
| P | 6978.251 | <0.001 | 122 | 9912.961 |
| C | 13642.806 | <0.001 | 67 | 16687.516 |
| t | 7561.4 | <0.001 | 133 | 10474.11 |
| tA | 16252.457 | <0.001 | 134 | 19163.167 |
| tP | 7779.286 | <0.001 | 134 | 10689.996 |
| tC | 16622.286 | <0.001 | 134 | 19532.996 |
| Constant only | 16624.032 | <0.001 | 135 | 19532.742 |

# the sub-model codes are the same as in Table 2.

Abbreviations: LR: likelihood-ratio. d.f.: degree of freedom. AIC: Akaike information criterion.

**Table 5 Fitted APC results of woman AIDS cases in the local population**

| Model^#^ | Increase of deviance | p-value of LR tests | Decrease in d.f. | AIC |
| --- | --- | --- | --- | --- |
| APC | NA | NA | NA | 2794.758 |
| Sub-models |  |  |  |  |
| AP | 1111.663 | <0.001 | 67 | 3082.799 |
| AC | 783.974 | 0.001 | 12 | 2865.11 |
| PC | 1178.425 | <0.001 | 54 | 3175.561 |
| Ad | 1172.907 | <0.001 | 79 | 3120.043 |
| Pd | 2127.374 | <0.001 | 121 | 3990.51 |
| Cd | 1243.125 | <0.001 | 66 | 3216.261 |
| A | 2179.039 | <0.001 | 80 | 4124.174 |
| P | 2364.314 | <0.001 | 122 | 4225.45 |
| C | 2471.423 | <0.001 | 67 | 4442.559 |
| t | 2191.576 | <0.001 | 133 | 4030.712 |
| tA | 3236.63 | <0.001 | 134 | 5073.766 |
| tP | 2429.483 | <0.001 | 134 | 4266.619 |
| tC | 3436.765 | <0.001 | 134 | 5273.901 |
| Constant only | 3532.366 | <0.001 | 135 | 5367.502 |

# the sub-model codes are the same as in Table 2.

Abbreviations: LR: likelihood-ratio. d.f.: degree of freedom. AIC: Akaike information criterion.

**Table 6 Fitted APC results of man AIDS cases in the immigrant population**

| Model^#^ | Increase of deviance | p-value of LR tests | Decrease in d.f. | AIC |
| --- | --- | --- | --- | --- |
| Full APC model | NA | NA | NA | 1958.793 |
| Sub-models |  |  |  |  |
| AP | 494.499 | <0.001 | 43 | 2013.957 |
| AC | 414.373 | <0.001 | 8 | 2003.831 |
| PC | 1334.764 | <0.001 | 34 | 2872.222 |
| Ad | 557.483 | <0.001 | 51 | 2060.941 |
| Pd | 6500.766 | <0.001 | 77 | 7952.224 |
| Cd | 1400.923 | <0.001 | 42 | 2922.381 |
| A | 1508.458 | <0.001 | 52 | 3009.916 |
| P | 6705.052 | <0.001 | 78 | 8154.51 |
| C | 2409.479 | <0.001 | 43 | 3928.937 |
| t | 6565.393 | <0.001 | 85 | 8000.851 |
| tA | 7676.187 | <0.001 | 86 | 9109.645 |
| tP | 6770.134 | <0.001 | 86 | 8203.592 |
| tC | 7478.247 | <0.001 | 86 | 8911.705 |
| Constant only | 7859.284 | <0.001 | 87 | 9290.742 |

# the sub-model codes are the same as in Table 2.

Abbreviations: LR: likelihood-ratio. d.f.: degree of freedom. AIC: Akaike information criterion.

**Table 7 Fitted APC results of woman AIDS cases in the immigrant population**

| Model^#^ | Increase of deviance | p-value of LR tests | Decrease in d.f. | AIC |
| --- | --- | --- | --- | --- |
| Full APC model | NA | NA | NA | 1474.728 |
| Sub-models |  |  |  |  |
| AP | 423.87 | <0.001 | 43 | 1533.696 |
| AC | 303.706 | 0.158 | 8 | 1483.532 |
| PC | 555.502 | <0.001 | 34 | 1683.328 |
| Ad | 449.519 | <0.001 | 51 | 1543.345 |
| Pd | 1728.412 | <0.001 | 77 | 2770.237 |
| Cd | 580.487 | <0.001 | 42 | 1692.313 |
| A | 555.136 | <0.001 | 52 | 1646.962 |
| P | 1813.35 | <0.001 | 78 | 2853.176 |
| C | 668.806 | <0.001 | 43 | 1778.632 |
| t | 1753.903 | <0.001 | 85 | 2779.729 |
| tA | 1888.525 | <0.001 | 86 | 2912.351 |
| tP | 1838.622 | <0.001 | 86 | 2862.448 |
| tC | 1846.264 | <0.001 | 86 | 2870.09 |
| Constant only | 1967.046 | <0.001 | 87 | 2988.872 |

# the sub-model codes are the same as in Table 2.

Abbreviations: LR: likelihood-ratio. d.f.: degree of freedom. AIC: Akaike information criterion.

**A.5 APC analysis of AIDS cases of the whole, local, and immigrant populations based on different transmitted routes in Zhejiang,China**

**Table 8 Fitted APC results of homosexual transmission AIDS cases in the the whole population**

| Model^#^ | Increase of deviance | p-value of LR tests | Decrease in d.f. | AIC |
| --- | --- | --- | --- | --- |
| Full APC model | NA | NA | NA | 3405.908 |
| Sub-models |  |  |  |  |
| AP | 879.959 | 0.004 | 68 | 3457.722 |
| AC | 2048.435 | <0.001 | 13 | 4736.197 |
| PC | 3248.795 | <0.001 | 54 | 5854.557 |
| Ad | 2302.559 | <0.001 | 81 | 4854.321 |
| Pd | 10493.803 | <0.001 | 122 | 12963.565 |
| Cd | 4787.683 | <0.001 | 67 | 7367.445 |
| A | 9297.699 | <0.001 | 82 | 11847.462 |
| P | 11904.719 | <0.001 | 123 | 14372.481 |
| C | 13333.419 | <0.001 | 68 | 15911.181 |
| t | 12029.747 | <0.001 | 135 | 14473.509 |
| tA | 19706.484 | <0.001 | 136 | 22148.246 |
| tP | 13423.816 | <0.001 | 136 | 15865.578 |
| tC | 17974.221 | <0.001 | 136 | 20415.983 |
| Constant only | 20767.471 | <0.001 | 137 | 23207.234 |

# the sub-model codes are the same as in Table 2.

Abbreviations: LR: likelihood-ratio. d.f.: degree of freedom. AIC: Akaike information criterion.

**Table 9 Fitted APC results of heterosexual transmission AIDS cases in the the whole population**

| Model^#^ | Increase of deviance | p-value of LR tests | Decrease in d.f. | AIC |
| --- | --- | --- | --- | --- |
| Full APC model | NA | NA | NA | 4367.004 |
| Sub-models |  |  |  |  |
| AP | 1804.898 | <0.001 | 68 | 5168.06 |
| AC | 1615.722 | <0.001 | 13 | 5088.884 |
| PC | 2749.386 | <0.001 | 54 | 6140.548 |
| Ad | 2523.643 | <0.001 | 81 | 5860.805 |
| Pd | 7178.916 | <0.001 | 122 | 10434.078 |
| Cd | 3471.896 | <0.001 | 67 | 6837.058 |
| A | 9820.782 | <0.001 | 82 | 13155.944 |
| P | 8252.218 | <0.001 | 123 | 11505.38 |
| C | 12212.199 | <0.001 | 68 | 15575.361 |
| t | 7948.304 | <0.001 | 135 | 11177.466 |
| tA | 15451.928 | <0.001 | 136 | 18679.09 |
| tP | 9020.913 | <0.001 | 136 | 12248.075 |
| tC | 16585.416 | <0.001 | 136 | 19812.578 |
| Constant only | 16868.996 | <0.001 | 137 | 20094.158 |

# the sub-model codes are the same as in Table 2.

Abbreviations: LR: likelihood-ratio. d.f.: degree of freedom. AIC: Akaike information criterion.

**Table 10 Fitted APC results of AIDS cases transmitted through other routes in the the whole population**

| Model^#^ | Increase of deviance | p-value of LR tests | Decrease in d.f. | AIC |
| --- | --- | --- | --- | --- |
| Full APC model | NA | NA | NA | 2351.556 |
| Sub-models |  |  |  |  |
| AP | 888.891 | 0.002 | 68 | 2336.845 |
| AC | 815.772 | 0.005 | 13 | 2373.726 |
| PC | 1008.698 | <0.001 | 54 | 2484.652 |
| Ad | 936.623 | <0.001 | 81 | 2358.577 |
| Pd | 1504.57 | <0.001 | 122 | 2844.524 |
| Cd | 1055.515 | <0.001 | 67 | 2505.469 |
| A | 1242.995 | <0.001 | 82 | 2662.949 |
| P | 1506.644 | <0.001 | 123 | 2844.597 |
| C | 1419.784 | <0.001 | 68 | 2867.738 |
| t | 1552.419 | <0.001 | 135 | 2866.373 |
| tA | 1872.723 | <0.001 | 136 | 3184.677 |
| tP | 1554.375 | <0.001 | 136 | 2866.329 |
| tC | 1875.268 | <0.001 | 136 | 3187.222 |
| Constant only | 1878.631 | <0.001 | 137 | 3188.585 |

# the sub-model codes are the same as in Table 2.

Abbreviations: LR: likelihood-ratio. d.f.: degree of freedom. AIC: Akaike information criterion.

**Table 11 Fitted APC results of homosexual transmission AIDS cases in the the local population**

| Model^#^ | Increase of deviance | p-value of LR tests | Decrease in d.f. | AIC |
| --- | --- | --- | --- | --- |
| Full APC model | NA | NA | NA | 2825.635 |
| Sub-models |  |  |  |  |
| AP | 724.269 | 0.397 | 67 | 2844.229 |
| AC | 1260.942 | <0.001 | 12 | 3490.902 |
| PC | 2073.717 | <0.001 | 54 | 4219.677 |
| Ad | 1400.11 | <0.001 | 79 | 3496.07 |
| Pd | 5929.258 | <0.001 | 121 | 7941.218 |
| Cd | 2775.248 | <0.001 | 66 | 4897.208 |
| A | 6065.569 | <0.001 | 80 | 8159.529 |
| P | 6945.734 | <0.001 | 122 | 8955.694 |
| C | 8008.285 | <0.001 | 67 | 10128.245 |
| t | 6642.525 | <0.001 | 133 | 8630.485 |
| tA | 11205.289 | <0.001 | 134 | 13191.25 |
| tP | 7652.887 | <0.001 | 134 | 9638.847 |
| tC | 10196.017 | <0.001 | 134 | 12181.977 |
| Constant only | 12005.643 | <0.001 | 135 | 13989.603 |

# the sub-model codes are the same as in Table 2.

Abbreviations: LR: likelihood-ratio. d.f.: degree of freedom. AIC: Akaike information criterion.

**Table 12 Fitted APC results of heterosexual transmission AIDS cases in the the local population**

| Model^#^ | Increase of deviance | p-value of LR tests | Decrease in d.f. | AIC |
| --- | --- | --- | --- | --- |
| Full APC model | NA | NA | NA | 3702.101 |
| Sub-models |  |  |  |  |
| AP | 1384.12 | <0.001 | 67 | 4229.809 |
| AC | 1116.39 | <0.001 | 12 | 4072.08 |
| PC | 1723.309 | <0.001 | 54 | 4594.999 |
| Ad | 1717.267 | <0.001 | 79 | 4538.956 |
| Pd | 4510.219 | <0.001 | 121 | 7247.909 |
| Cd | 2071.692 | <0.001 | 66 | 4919.381 |
| A | 6868.079 | <0.001 | 80 | 9687.769 |
| P | 5317.609 | <0.001 | 122 | 8053.299 |
| C | 7717.259 | <0.001 | 67 | 10562.948 |
| t | 4858.33 | <0.001 | 133 | 7572.019 |
| tA | 9831.929 | <0.001 | 134 | 12543.619 |
| tP | 5666.888 | <0.001 | 134 | 8378.577 |
| tC | 10622.051 | <0.001 | 134 | 13333.741 |
| Constant only | 10920.406 | <0.001 | 135 | 13630.095 |

# the sub-model codes are the same as in Table 2.

Abbreviations: LR: likelihood-ratio. d.f.: degree of freedom. AIC: Akaike information criterion.

**Table 13 Fitted APC results of AIDS cases transmitted through other routes in the the local population**

| Model^#^ | Increase of deviance | p-value of LR tests | Decrease in d.f. | AIC |
| --- | --- | --- | --- | --- |
| Full APC model | NA | NA | NA | 1781.372 |
| Sub-models |  |  |  |  |
| AP | 705.086 | 0.597 | 67 | 1732.199 |
| AC | 650.222 | 0.6 | 12 | 1787.336 |
| PC | 727.016 | 0.249 | 54 | 1780.13 |
| Ad | 733.686 | 0.424 | 79 | 1736.8 |
| Pd | 1002.206 | <0.001 | 121 | 1921.319 |
| Cd | 757.04 | 0.128 | 66 | 1786.154 |
| A | 1008.246 | <0.001 | 80 | 2009.36 |
| P | 1005.203 | <0.001 | 122 | 1922.316 |
| C | 1060.256 | <0.001 | 67 | 2087.369 |
| t | 1030.187 | <0.001 | 133 | 1925.301 |
| tA | 1293.121 | <0.001 | 134 | 2186.235 |
| tP | 1033.164 | <0.001 | 134 | 1926.278 |
| tC | 1299.904 | <0.001 | 134 | 2193.017 |
| Constant only | 1300.557 | <0.001 | 135 | 2191.67 |

# the sub-model codes are the same as in Table 2.

Abbreviations: LR: likelihood-ratio. d.f.: degree of freedom. AIC: Akaike information criterion.

**Table 14 Fitted APC results of homosexual transmission AIDS cases in the the immigrant population**

| Model^#^ | Increase of deviance | p-value of LR tests | Decrease in d.f. | AIC |
| --- | --- | --- | --- | --- |
| Full APC model | NA | NA | NA | 1720.492 |
| Sub-models |  |  |  |  |
| AP | 416.288 | <0.001 | 43 | 1728.684 |
| AC | 417.433 | <0.001 | 8 | 1799.829 |
| PC | 1023.776 | <0.001 | 34 | 2354.173 |
| Ad | 514.799 | <0.001 | 51 | 1811.195 |
| Pd | 4613.361 | <0.001 | 77 | 5857.757 |
| Cd | 1135.414 | <0.001 | 42 | 2449.81 |
| A | 1238.294 | <0.001 | 52 | 2532.69 |
| P | 5005.707 | <0.001 | 78 | 6248.103 |
| C | 1961.705 | <0.001 | 43 | 3274.101 |
| t | 4720.66 | <0.001 | 85 | 5949.056 |
| tA | 5573.198 | <0.001 | 86 | 6799.594 |
| tP | 5112.493 | <0.001 | 86 | 6338.889 |
| tC | 5346.372 | <0.001 | 86 | 6572.768 |
| Constant only | 5935.088 | <0.001 | 87 | 7159.484 |

# the sub-model codes are the same as in Table 2.

Abbreviations: LR: likelihood-ratio. d.f.: degree of freedom. AIC: Akaike information criterion.

**Table 15 Fitted APC results of heterosexual transmission AIDS cases in the the immigrant population**

| Model^#^ | Increase of deviance | p-value of LR tests | Decrease in d.f. | AIC |
| --- | --- | --- | --- | --- |
| Full APC model | NA | NA | NA | 1791.891 |
| Sub-models |  |  |  |  |
| AP | 520.327 | <0.001 | 43 | 1902.366 |
| AC | 336.315 | 0.012 | 8 | 1788.354 |
| PC | 747.805 | <0.001 | 34 | 2147.844 |
| Ad | 531.377 | <0.001 | 51 | 1897.417 |
| Pd | 3390.378 | <0.001 | 77 | 4704.417 |
| Cd | 760.795 | <0.001 | 42 | 2144.835 |
| A | 1002.64 | <0.001 | 52 | 2366.679 |
| P | 3419.747 | <0.001 | 78 | 4731.786 |
| C | 1172.266 | <0.001 | 43 | 2554.306 |
| t | 3401.033 | <0.001 | 85 | 4699.072 |
| tA | 3943.135 | <0.001 | 86 | 5239.174 |
| tP | 3430.452 | <0.001 | 86 | 4726.492 |
| tC | 3881.134 | <0.001 | 86 | 5177.173 |
| Constant only | 3966.549 | <0.001 | 87 | 5260.588 |

# the sub-model codes are the same as in Table 2.

Abbreviations: LR: likelihood-ratio. d.f.: degree of freedom. AIC: Akaike information criterion.

**Table 16 Fitted APC results of AIDS cases transmitted through other routes in the the immigrant population**

| Model^#^ | Increase of deviance | p-value of LR tests | Decrease in d.f. | AIC |
| --- | --- | --- | --- | --- |
| Full APC model | NA | NA | NA | 927.052 |
| Sub-models |  |  |  |  |
| AP | 343.718 | 0.128 | 43 | 915.676 |
| AC | 277.33 | 0.534 | 8 | 919.288 |
| PC | 376.759 | 0.004 | 34 | 966.717 |
| Ad | 353.256 | 0.119 | 51 | 909.214 |
| Pd | 699.048 | <0.001 | 77 | 1203.005 |
| Cd | 384.018 | 0.004 | 42 | 957.976 |
| A | 366.614 | 0.051 | 52 | 920.571 |
| P | 700.938 | <0.001 | 78 | 1202.895 |
| C | 392.765 | 0.002 | 43 | 964.723 |
| t | 708.018 | <0.001 | 85 | 1195.975 |
| tA | 724.555 | <0.001 | 86 | 1210.513 |
| tP | 709.96 | <0.001 | 86 | 1195.917 |
| tC | 721.983 | <0.001 | 86 | 1207.941 |
| Constant only | 726.213 | <0.001 | 87 | 1210.17 |

# the sub-model codes are the same as in Table 2.

Abbreviations: LR: likelihood-ratio. d.f.: degree of freedom. AIC: Akaike information criterion.
